# Dynamic Cascade Spiking Neural Network Supervisory Controller for a Nonplanar Twelve-Rotor UAV

**DOI:** 10.3390/s25041177

**Published:** 2025-02-14

**Authors:** Cheng Peng, Guanyu Qiao, Bing Ge

**Affiliations:** Changchun Institute of Optics, Fine Mechanics and Physics, Chinese Academy of Sciences, Changchun 130033, China

**Keywords:** nonplanar twelve-rotor UAV, intelligent composite controller, dynamic cascade spiking neural network, supervisory feedforward control

## Abstract

Unknown variables in the environment, such as wind disturbance during a flight, affect the accurate trajectory of multi-rotor UAVs. This study focuses on the intelligent supervisory neurocontrol of trajectory tracking for a nonplanar twelve-rotor UAV to address this issue. Firstly, a twelve-rotor UAV is developed with a nonplanar structure, which makes up for the defects of conventional multi-rotors with weak yaw movement. A characteristic model of the twelve-rotor UAV is devised so as to facilitate intelligent controller design without losing model information. For the purpose of achieving accurate and fast trajectory tracking and strong self-learning ability, an intelligent composite controller combining adaptive sliding-mode feedback control and dynamic cascade spiking neural network (DCSNN) supervisory feedforward control is proposed. The novel dynamic cascade network structure is constructed to better adapt to changing data and unstable environments. The weight learning algorithm and dynamic cascade structure learning algorithm work together to ensure network stability and robustness. Finally, comparative numerical simulations and twelve-rotor UAV prototype experiments verify the superior tracking control performance, even outdoors with wind disturbances.

## 1. Introduction

Multi-rotor unmanned aerial vehicles (UAVs) are widely used in various fields, such as military and industrial applications, due to their superior mobility and vertical takeoff and landing capabilities [1,2,3]. Among them, quad-rotor UAVs are a typical representative example. Subsequently, other types of multi-rotor UAVs have emerged, such as tri-rotor UAVs [4,5], eight-rotor UAVs [6,7,8], and hex-rotor UAVs [9,10]. These variations theoretically enhance the reliability of UAVs. The twelve-rotor UAV with a nonplanar structure proposed in this study makes up for the defect of conventional multi-rotors with weak yaw movement and has a higher drive capability, a greater payload capacity, and better damage tolerance than quad-rotor UAVs.

With the popularization of multi-rotor UAV applications, the complex and variable flight environment brings challenges in trajectory-tracking control. Meanwhile, multi-rotor UAVs have the inherent characteristics of extreme nonlinearity, strong coupling, and open-loop instability with multiple input variables and multiple output variables. Therefore, the robust trajectory-tracking control problem of multi-rotor UAVs has been increasingly gaining attention. Scholars have proposed a variety of effective solutions, such as PID control, backstepping control, active disturbance rejection control, sliding-mode control, and predictive control [11,12,13,14]. In response to the nonlinear characteristics of multi-rotor UAVs, it is common to linearize the system around the equilibrium point and then design corresponding linear feedback control laws [15]. Since the linearization method around the equilibrium point cannot adapt to strong maneuvering control situations, it is necessary to design nonlinear control laws. In ref. [16], a trajectory-tracking controller for a multi-rotor UAV was designed using the nonlinear PID control method, and stable tracking of the expected trajectory was realized. In ref. [17], an adaptive mixing controller was formulated to deal with a linear parameter-varying system and provide robustness against disturbances. In ref. [18], a Fuzzy-LADRC was proposed to improve the dynamic performance of a quad-rotor system. It made use of the adaptive ability of the fuzzy controller and the anti-interference ability of LADRC. Another study [19] utilized a fuzzy logic system to approximate the nonlinear relationship of a quad-rotor UAV system and a disturbance observer was designed to compensate for composite disturbances. The simulation results demonstrated the validity of the proposed control strategy. The authors of [20] proposed a state-space LS-SVM disturbance observer that estimated the disturbance and quadrotor system states simultaneously. It provided robust state observation.

Considering that composite control can further improve the dynamic response performance of a multi-rotor UAV control system in variable flight environments, a series of studies on the composite control of multi-rotor UAVs have been conducted. One systematic design methodology used a centralized feedforward/feedback control architecture based on the H∞ norm as a measure for the design criteria, and it was applied to the trajectory-tracking control of a tri-rotor UAV and a quad-rotor UAV [21]. In ref. [22], a velocity-free desired compensation trajectory-tracking strategy for quad-rotors was presented, integrating an adaptive robust integral of the sign of the RISE feedback mechanism concerning parametric uncertainties and external disturbances. Model-based feedforward control relying on the expected trajectory was devised to achieve accurate model compensation. Due to the adaptive, self-organizing, and self-learning characteristics of artificial neural networks, many scholars use them as feedforward controllers to achieve composite control. In ref. [23], a neural adaptive integral robust controller was proposed, and a feedforward controller with minimal learning parameters was used to estimate unknown disturbances. The estimated residual was suppressed by a robust feedback controller to obtain high-precision tracking performance. The authors of [24] studied the feedforward control algorithm of a cerebellar model articulation network to achieve adaptive optimal tracking control. In ref. [25], the composite control strategy of a dual feedforward/feedback architecture was proposed, and the artificial neural network, as the expected module, realized feedforward control through adaptive learning.

Recent advances in artificial intelligence have sparked significant interest in spiking neural networks (SNNs) as a new type of neural network [26,27,28]. Spiking neurons are employed to convey and transmit spatiotemporal information with pulse coding, as real biological neurons do [29]. Their processing power and efficiency are significantly superior to those of traditional neural networks, making them a more reasonable model. Currently, SNNs have yielded notable results in pattern recognition, intelligent computation, and brain-like research. SNN studies in control systems have shown promise [30]. In particular, more extensive applications of SNNs to robotic control problems have emerged. In ref. [31], the combination of an SNN and a CNN realized the motion control of a two-DOF robot. In ref. [32], a hybrid learning control system implemented on an SNN platform based on the Izhkevich model of a single neuron was tested on a two-wheeled mobile robot and a four-link manipulator robot to verify its validity. The study in [33] proposed an autonomous learning model for robots based on an SNN that could complete specific tasks of autonomous learning. In ref. [34], based on the principles of the neural engineering framework, a spiking PID controller was designed and simulated for a three-DOF robotic arm. The results demonstrated good accuracy and efficiency in the designated trajectories.

Therefore, this study proposes a novel DCSNN supervisory controller based on the idea of the feedforward control of neural networks that addresses the problem of accurate trajectory tracking and improves the intelligent self-learning ability of UAVs in the face of a changing flight environment. The novel DCSNN is more flexible and enjoys some distinguished advantages, such as adaptability to changing data and unstable environments. In addition, the DCSNN is able to overcome issues that require prior knowledge of network structure information and the sample proportion distribution in the SNN. A composite control framework comprising an adaptive sliding-mode algorithm as the feedback controller and the DCSNN as the supervised feedforward controller is established for a nonplanar twelve-rotor UAV. A characteristic model of the twelve-rotor is created by using the characteristic model method. The characteristic model theory was proposed in the 1980s. It can achieve equivalence between a coupled nonlinear system and an input-decoupled time-varying differential model without losing model information [35]. Then, the stability of the closed-loop composite control system based on the characteristic model of the twelve-rotor UAV is analyzed. Comparative numerical simulations and prototype experiments are carried out on the twelve-rotor UAV to confirm that the proposed composite strategy has more accurate trajectory-tracking control performance in a windy outdoor environment.

## 2. Flight Principle and Mathematical Model

**Figure 1:**
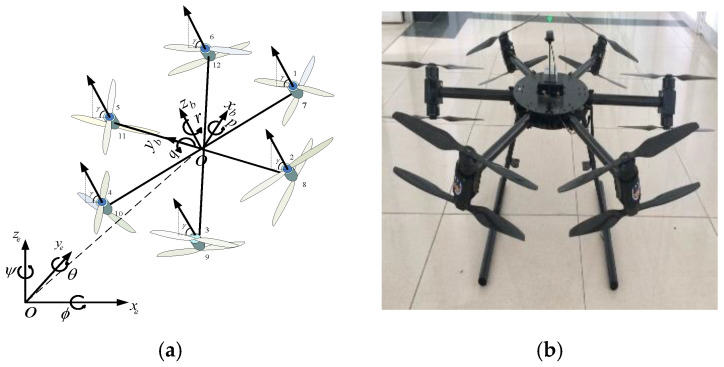
The scheme of the nonplanar twelve-rotor UAV. (**a**) Schematic diagram; (**b**) physical image.

Figure 1 presents the structure of the nonplanar twelve-rotor UAV, in which six equal-length lightweight connecting rods that are used as arms are distributed around the center of the body to form the horizontal plane of the body. The angle between the arms is 60 degrees. Rotors are installed in pairs at the end of each connecting rod. Rotors 2, 4, 6, 7, 9, and 11 rotate clockwise, while rotors 1, 3, 5, 8, 10, and 12 rotate counterclockwise. The angle between each rotor axis and the body is set to γ(0<γ≤30), and each of the two adjacent rotor axes points in opposite directions. The attitude and position movement of the twelve-rotor UAV is realized by changing the rotor speed. Compared with conventional quadrotors, the thrust and torque generated by the rotors do not act only in a single motion channel. Due to the nonplanar design of the structure, the component of thrust increases in the yaw direction, which results in the fact that the yaw control torque, is significantly enhanced, and yaw motion is no longer generated by weak anti-torque, as in a conventional UAV.

The rotational dynamic model can be denoted as follows:

(1) $$\left\{\begin{array}{c} I_{x}\dot{p}=M_{x}-\left(I_{z}-I_{y}\right)rq\\ I_{y}\dot{q}=M_{y}-\left(I_{x}-I_{z}\right)pr\\ I_{z}\dot{r}=M_{z}-\left(I_{y}-I_{z}\right)qp \end{array}\right.$$

where $\omega ={\left[p,q,r\right]}^{T}$ expresses the angular velocity, $I_{x},I_{y},I_{z}$ are the moments of inertia of each axis, and $M={\left[M_{x},M_{y},M_{z}\right]}^{T}$ is the projection of the resultant torque acting on the twelve-rotor UAV in the body coordinate system.

The translational dynamic model based on the Newton–Euler formula is derived as follows:

(2) $$\left[\begin{array}{c} {\ddot{P}}_{x}\\ {\ddot{P}}_{y}\\ {\ddot{P}}_{z} \end{array}\right]=\frac{1}{m}\left[\begin{array}{c} \begin{array}{c} F_{x}\cos\psi \cos\theta +F_{y}\left(\cos\psi \sin\theta \cos\phi -\sin\psi \cos\phi \right)\\ +F_{z}\left(\cos\psi \sin\theta \cos\phi +\sin\psi \sin\phi \right)\\ F_{x}\sin\psi \cos\theta +F_{y}\left(\cos\psi \sin\theta \sin\phi +\cos\psi \cos\phi \right)\\ +F_{z}\left(\sin\psi \sin\theta \cos\phi -\cos\psi \sin\phi \right)\\ -F_{x}\sin\theta +F_{y}\sin\phi \cos\psi +F_{z}\cos\theta \cos\phi -mg \end{array} \end{array}\right]$$

where $P={\left[P_{x},P_{y},P_{z}\right]}^{T}$ expresses the translational position. The Euler angles $\eta ={\left[\phi ,\theta ,\psi \right]}^{T}$ define the attitude, g is the acceleration of gravity, m is the mass of the twelve-rotor UAV, and $F={\left[\begin{array}{ccc} F_{x} & F_{y} & F_{z} \end{array}\right]}^{T}$ describes the resultant force. More modeling details can be found in our previous article [35]; a longer description is not provided for the sake of simplicity.

## 3. Characteristic Model of the Twelve-Rotor UAV

Through the mathematical model established in the previous section, it can be clearly seen that the twelve-rotor UAV is a complex system with strong coupling and nonlinearity, which increases the difficulty of the controller design. Hence, characteristic model theory is applied in this study. According to the characteristic relationship between the control variable and the output variable, a characteristic model of the twelve-rotor UAV is established to simplify the dynamic model without losing high-order information, which lays the foundation for subsequent intelligent control.

In the general case of small attitude angles in the twelve-rotor UAV, from Equation (2), the trajectory error equation can be deduced as follows:

(3) $$\left\{\begin{array}{c} {\ddot{P}}_{ex}=-\frac{1}{m}\sin\gamma F_{e}cos\psi \sin\theta \cos\phi +{\ddot{P}}_{rx}\\ {\ddot{P}}_{ey}=-\frac{1}{m}\sin\gamma F_{e}\cos\psi \sin\phi +{\ddot{P}}_{ry}\\ {\ddot{P}}_{ez}=-\frac{1}{m}\sin\gamma F_{e}cos\theta \cos\phi +g+{\ddot{P}}_{rz} \end{array}\right.$$

where $P_{e}={\left[P_{ex},P_{ey},P_{ez}\right]}^{T}$ describes the trajectory errors in three directions, $P_{r}={\left[P_{rx},P_{ry},P_{rz}\right]}^{T}$ represents the desired trajectory in three directions, $P=P_{r}-P_{e}$, and $F_{e}=2k\sum_{i=1}^{6}\Omega_{i}^{2}$ expresses the thrust force perpendicular to the direction of the rotor, with k as a thrust coefficient.

The state equation is expressed by taking the fourth derivative of Equation (3) as follows:

(4) $$x^{\left(4\right)}=A\ddot{x}+B_{0}(x)u+B_{1}\dot{u}+B_{2}\ddot{u}+C$$

where $x=P_{e}$ denotes the model output, and the controlling quantity is given by $u={\left[F_{e},M_{x},M_{y},M_{z}\right]}^{T}$.

$\begin{array}{c} A=\left[\begin{array}{ccc} -{\left(\dot{\psi }\right)}^{2}+\tan\psi \tan\phi \cdot \dot{\psi }\dot{\phi }+\frac{\tan\psi }{I_{z}}(I_{y}-I_{x})qp-\frac{I_{x}-I_{z}}{I_{y}}pr & -\sin\theta \cdot \dot{\theta }\dot{\phi } & -\sin\psi \cdot \dot{\psi }\dot{\theta }\\ \frac{2\tan\psi }{\sin\theta }\dot{\psi }\dot{\phi } & -{\left(\dot{\psi }\right)}^{2}+{\left(\dot{\phi }\right)}^{2}-\frac{\tan\psi }{I_{z}}(I_{y}-I_{x})pq & -\frac{I_{z}-I_{y}}{I_{x}}pr\\ \frac{(I_{x}-I_{z})pr}{I_{y}\cos\psi } & -\frac{2\sin\theta \cdot \dot{\theta }\dot{\phi }}{\cos\psi } & {\left(\dot{\theta }\right)}^{2}-\tan\phi {\left(\dot{\phi }\right)}^{2}+\frac{(I_{z}-I_{y})qr\tan\phi }{I_{x}} \end{array}\right],\\ B_{0}(x)=\left[\begin{array}{llll} 0 & -\frac{{\ddot{x}}_{1}}{I_{x}} & \frac{{\ddot{x}}_{1}}{I_{y}} & \frac{\tan\psi }{I_{z}}{\ddot{x}}_{1}\\ 0 & -\frac{{\ddot{x}}_{3}+g}{I_{x}} & 0 & -\frac{\tan\psi }{I_{z}}{\ddot{x}}_{2}\\ 0 & -\frac{{\ddot{x}}_{3}+g}{I_{x}}\tan\phi & -\frac{{\ddot{x}}_{1}}{\cos\psi I_{y}} & 0 \end{array}\right],\\ B_{1}=\left[\begin{array}{llll} \frac{2}{m}\sin\gamma (-\sin\psi \dot{\psi }\sin\theta \cos\phi +\cos\psi \sin\theta \dot{\theta }\cos\phi ) & 0 & 0 & 0\\ \frac{2}{m}\sin\gamma (\sin\psi \dot{\psi }\sin\phi -\cos\psi \cos\phi \dot{\phi }) & 0 & 0 & 0\\ \frac{2}{m}\sin\gamma (-\sin\theta \dot{\theta }\cos\phi -\cos\theta \sin\phi \dot{\phi }) & 0 & 0 & 0 \end{array}\right],\\ B_{2}=\left[\begin{array}{llll} \frac{1}{m}\sin\gamma sin\theta cos\psi cos\phi & 0 & 0 & 0\\ -\frac{1}{m}\sin\gamma \cos\psi \sin\phi & 0 & 0 & 0\\ \frac{1}{m}\sin\gamma cos\theta \cos\phi & 0 & 0 & 0 \end{array}\right],\\ C=\left[\begin{array}{c} -\sin\psi \dot{\psi }\dot{\theta }g++P_{rx}^{\left(4\right)}\\ -\frac{(I_{z}-I_{y})qrg}{I_{x}}++P_{ry}^{\left(4\right)}\\ g{\left(\dot{\theta }\right)}^{2}-g\tan\phi {\left(\dot{\phi }\right)}^{2}+\frac{g\tan\phi (I_{z}-I_{y})qr}{I_{x}}+P_{rz}^{\left(4\right)} \end{array}\right]. \end{array}$

Further, we have

(5) $$x_{j}^{\left(4\right)}=A_{j}\ddot{x}+\sum_{s=0}^{2}\sum_{l=1}^{4}b_{s,jl}(x)u_{l}^{\left(s\right)}+C_{j},\;j=1,2,3$$

where $A_{j}$ is a row vector of A, and $B_{s}(x)={\left(b_{s,ij}(x)\right)}_{3\times 4},s=0,1,2,i=1,2,3$. Then, adding ${\dot{x}}_{j}$ to both sides of Equation (5), we have

(6) $${\dot{x}}_{j}=\sum_{l=1}^{4}b_{0}(x)u_{l}+K_{j}(t)$$

where $K_{j}(t)=-x_{j}^{\left(4\right)}+A_{j}\ddot{x}+\sum_{s=1}^{2}\sum_{l=1}^{4}b_{s,jl}(x)u_{l}^{\left(s\right)}+C_{j}+{\dot{x}}_{j}$. Taking the derivative of Equation (6), we have

(7) $${\ddot{x}}_{j}=\sum_{l=1}^{4}\frac{db_{0}(x)}{dt}u_{l}+\sum_{l=1}^{4}b_{0}(x){\dot{u}}_{l}+\frac{dK_{j}(t)}{dt}$$

Equations (6) and (7) are discretized using the difference method; then, adding them together, we have

(8) $$x_{j}(k+1)=f_{j1}(k)x_{j}(k)+f_{j2}(k)x_{j}(k-1)+\sum_{l=1}^{4}g_{jl}(k)u_{l}(k)+\sum_{l=1}^{4}g_{j,p+l}(k)u_{l}(k-1)+W_{j}(k)$$

where $f_{j1}(k)=2-T,f_{j2}(k)=T-1,g_{jl}(k)=T^{2}b_{0}(x(k))+2Tb_{0}(x(k))-Tb_{0}(x(k-1)),$ with T as the sampling period,$g_{j,p+l}(k)=-Tb_{0}(x(k)),W_{j}(k)=(T^{2}+T)K(k)-TK(k-1),$ and $\left|W_{j}(k)\right|\leq 2M_{j}T+M_{j}T^{2}$ with $M_{j}$ as a positive number; then, we have $\underset{T\to 0^{+}}{\lim}\left|W_{j}(k)\right|=0$. It can be noted that the model error is less than a given limit under the condition of a small sampling period.

The characteristic model of the twelve-rotor UAV can be depicted as follows:

(9) $$x(k+1)=F_{1}(k)x(k)+F_{2}(k)x(k-1)+G_{0}(k)u(k)+G_{1}(k)u(k-1)$$

where $F_{1}(k),F_{2}(k),G_{0}(k),G_{1}(k)$ are characteristic parameters of the twelve-rotor UAV equipped with slow time-varying properties, and x(k+1) presents the discrete trajectory error in three directions at time k+1. In the same way, x(k) is at time k, and x(k−1) is at time k−1. u(k) is the discrete controlling quantity at time k. In general, $G_{1}(k)=0$ when the relative degree of the system is greater than or equal to 2 [36]. As a result, the characteristic model is

(10) $$x(k+1)=F_{1}(k)x(k)+F_{2}(k)x(k-1)+G_{0}(k)u(k)$$

## 4. Intelligent Composite Control of the Twelve-Rotor UAV

**Figure 2:**
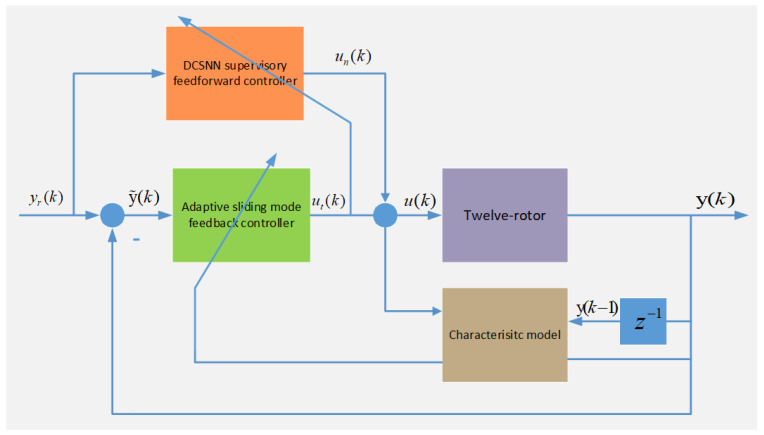
The composite control framework.

In the face of a complex and variable flight environment, such as one involving wind disturbances, an intelligent composite control strategy is proposed, with an adaptive sliding-mode algorithm as the feedback controller and a new DCSNN as the supervised feedforward controller. The composite control framework is shown in Figure 2. It improves the dynamic response performance and self-learning ability of the twelve-rotor control system, and the system disturbance can be suppressed in a timely manner without waiting for feedback errors in the system, which allows high-precision trajectory-tracking control.

### 4.1. Adaptive Feedback Controller Design

On the basis of the characteristic model shown in Equation (10), the adaptive sliding-mode feedback controller is designed to provide trajectory-tracking stability. Firstly, the recursive least-square algorithm with a forgetting factor is applied to estimate the characteristic parameter $G_{0}(k)$ as follows:

(11) $$\left\{\begin{array}{c} \begin{array}{l} {\hat{G}}_{0}(k)={\hat{G}}_{0}(k-1)+L(k)[x(k)-F_{1}(k)x(k-1)\\ +F_{2}(k)x(k-2)-u^{T}(k){\hat{G}}_{0}(k-1)] \end{array}\\ L(k)=Q(k-1)u(k){\left[\lambda +u^{T}(k)Q(k-1)u(k)\right]}^{-1}\\ Q(k)=\frac{1}{\lambda }\left[I-L(k)u^{T}(k)\right]Q(k-1) \end{array}\right.$$

where Q(k) and L(k), respectively, describe the error covariance matrix and gain matrix, and λ denotes the forgetting factor. Then, the characteristic identification model can be obtained as follows:

(12) $$x(k+1)={\hat{F}}_{1}(k)x(k)+{\hat{F}}_{2}(k)x(k-1)+{\hat{G}}_{0}(k)u(k)+\Delta (k)$$

where ${\hat{F}}_{1}(k)=(2-T)\cdot I$, ${\hat{F}}_{2}(k)=(T-1)\cdot I$, and Δ(k) expresses the total disturbances, including the identification error, uncertainties, and external disturbances, where $\left|\Delta (k)\right|<\tau_{m}$, with $\tau_{m}$ as a positive constant.

Then, on the basis of the characteristic identification model, the discrete adaptive sliding-mode feedback controller $u_{t}$ is designed to ensure stable trajectory tracking in the twelve-rotor system. Combining the adaptive control law $u_{ap}$ and the sliding-mode control law $u_{sl}$ devised as follows, buffeting is effectively weakened while maintaining the rapid convergence of the system. In addition, compared with some complex reaching law designs, the sliding-mode reaching law in this study is easy to realize and apply in practical flight missions.

(13) $$u_{t}=u_{ap}+u_{sl}$$

with

(14) $$u_{ap}=-\frac{1}{{\hat{G}}_{0}(k)}[{\hat{F}}_{1}(k)x(k)+{\hat{F}}_{2}(k)x(k-1)]$$

(15) $$u_{sl}=\frac{1}{{\hat{G}}_{0}(k)}[(1-qT)x(k)-\varepsilon T\left|x(k)\right|arctan(x(k))]$$

where q and ε are approaching term constants, and T is the sampling period, satisfying

(16) $$q>0,\;\varepsilon >\frac{\tau_{m}}{T\left|x_{0}(k)\right|\arctan\left|x_{0}(k)\right|},\;0<T<\frac{1}{q+\pi \varepsilon }$$

where $x_{0}(k)$ is the designed minimal trajectory-tracking error.

Defining $\kappa =\frac{x(k+1)}{x(k)}$, we have $\kappa =1-qT-\varepsilon T\arctan\left|x(k)\right|+\frac{\Delta (k)}{x(k)}$. When κ=1, $\Delta (k)=qTx_{0}(k)+\varepsilon Tx_{0}(k)\arctan\left|x_{0}(k)\right|$. Since $x_{0}(k)$ has converged to the origin, $\arctan\left[x_{0}(k)\right]\approx x_{0}(k)$ is considered. We have $\Delta (k)=qTx_{0}(k)+\varepsilon Tx_{0}(k)\left|x_{0}(k)\right|$. Then, $\left|qTx_{0}(k)+\varepsilon Tx_{0}(k)\left|x_{0}(k)\right|\right|<\tau_{m}$. In the case of $x_{0}(k)>0$, it is found that $\varepsilon Tx_{0}^{2}(k)+qTx_{0}(k)-\tau_{m}<0$. Then, $0<x_{0}(k)<\frac{-qT+\sqrt{q^{2}T^{2}+4\varepsilon T\tau_{m}}}{2\varepsilon T}$. In the case of $x_{0}(k)<0$, we have $\varepsilon Tx_{0}^{2}(k)-qTx_{0}(k)-\tau_{m}<0$. Then, $\frac{-qT+\sqrt{q^{2}T^{2}+4\varepsilon T\tau_{m}}}{2\varepsilon T}<x_{0}(k)<0$. As a consequence, when the parameters satisfy Equation (21), the feedback tracking error can be limited to $\left|x_{0}(k)\right|<\frac{-qT+\sqrt{q^{2}T^{2}+4\varepsilon T\tau_{m}}}{2\varepsilon T}$.

### 4.2. DCSNN Supervisory Feedforward Controller Design

#### 4.2.1. Network Structure Framework

**Figure 3:**
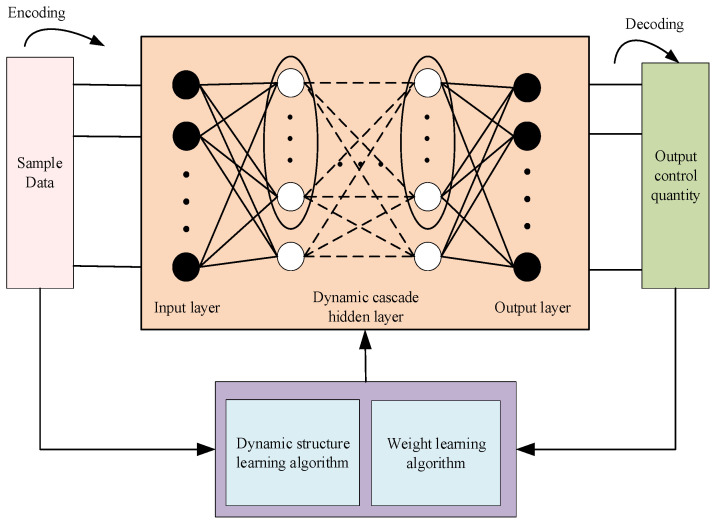
The DCSNN structure framework.

The DCSNN supervisory feedforward controller is devised to further improve the dynamic response performance of the control system. The novel dynamic cascade network structure is able to increase the ability to adapt to varied and unstable environments, as shown in Figure 3. The desired tracking positions and the output of the feedback controller are used as the sample data. The Poisson coding method is applied as a data encoder. The leaky integrate-and-fire (LIF) model is expressed as

(17) $$C_{m}\frac{dV(t)}{dt}=-\tau_{L}(V(t)-U_{res})+I(t)$$

where $C_{m}$ represents the membrane capacitance, V(t) and $U_{res}$ express the membrane potential and leak reversal potential, $\tau_{L}$ describes the leak conductance, and I(t) denotes the aggregate synaptic current. A spike is issued in the case of V(t) exceeding the threshold $V_{T}$, and then V(t) is reset to $U_{res}$. When the latest spike is issued at time $t_{last}$, $V(t_{last})=U_{res}$.

**Figure 4:**
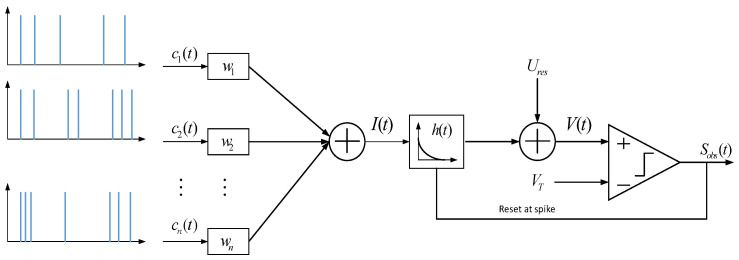
The LIF neuron model.

The LIF neuron model is depicted in Figure 4. The neuron receives inputs from n synapses, and $t_{i}^{1},t_{i}^{2},\ldots ,t_{i}^{n_{i}}$ express the times of spikes’ arrival at the $i^{th}$ synapse. The input signal of the $i^{th}$ synapse is obtained as follows:

(18) $$c_{i}(t)=\sum_{f=1}^{n_{i}}\alpha (t-t_{i}^{f})$$

The synaptic current received by the neuron is rewritten as

(19) $$I(t)=w^{T}c(t)$$

where the weight vector $w={\left[w_{1},w_{2},\ldots w_{n}\right]}^{T}$ represents the synaptic weights and strengths, and $c(t)={\left[c_{1}(t),c_{2}(t),\ldots c_{n}(t)\right]}^{T}$; then, the leaky impulse response h(t) is taken as follows:

(20) $$h(t)=\frac{1}{C_{m}}\exp(-t/\lambda_{L})u(t)$$

with $\lambda_{L}=C_{m}/\tau_{L}$. Then, we can have

(21) $$\begin{array}{ll} V(t) & =U_{res}+(I(t)u(t-t_{last}))\cdot h(t)\\ & =U_{res}+\sum_{i=1}^{n}w_{i}[c_{i}(t)u(t-t_{last})\cdot h(t)]\\ & =U_{res}+w^{T}d(t) \end{array}$$

where $d(t)={\left[d_{1}(t),d_{2}(t),\ldots d_{n}(t)\right]}^{T}$ with $d_{i}(t)=(c_{i}(t)u(t-t_{last}))\cdot h(t)$ carries all of the input information needed to determine the membrane potential.

#### 4.2.2. Supervised Weight Learning Algorithm

Further, a weight learning algorithm and a dynamic cascade structure learning algorithm are designed to work together. The aim of supervised weight learning is to determine the weight $w={\left[w_{1},w_{2},\ldots w_{n}\right]}^{T}$ for the neuron so as to reach the desired spike train $S_{des}(t)$, which is described as follows:

(22) $$S_{des}(t)=\sum_{i=1}^{f}\delta (t-t_{des}^{i})$$

The observed spike train is expressed as

(23) $$S_{obs}(t)=\sum_{i=1}^{f^{\prime }}\delta (t-t_{obs}^{i})$$

where $t_{obs}^{i}$ is the issued spike time of the neuron. The error function is defined as

(24) $$e(t)=S_{des}(t)-S_{obs}(t)$$

The cost function with respect to w is denoted as follows:

(25) $$J(w)=\frac{1}{2}\int_{0}^{T}\left|e(t)\right|{\left(V_{des}(t)-V(t)\right)}^{2}dt$$

with T as the training duration. Then, the desired weight is

(26) $$w_{des}=\underset{w}{\arg\min}J(w)$$

Due to the jump discontinuities of the weight space in Equation (25), for the sake of simplicity, the cost function is minimized independently at each time instant. Then, the supervised learning rule is devised as follows:

(27) $$\Delta w=r\int_{0}^{T}e(t)\frac{\hat{d}(t)}{\left\|\hat{d}(t)\right\|}$$

where r is a constant magnitude, ${\hat{d}}_{i}(t)=c_{i}(t)\cdot \hat{h}(t)$, and $\hat{h}(t)=1/C_{m}\exp(-t/{\lambda^{\prime }}_{L})u(t)$ with ${\lambda^{\prime }}_{L}<\lambda_{L}$.

#### 4.2.3. Dynamic Cascade Structure Learning Algorithm

**Figure 5:**
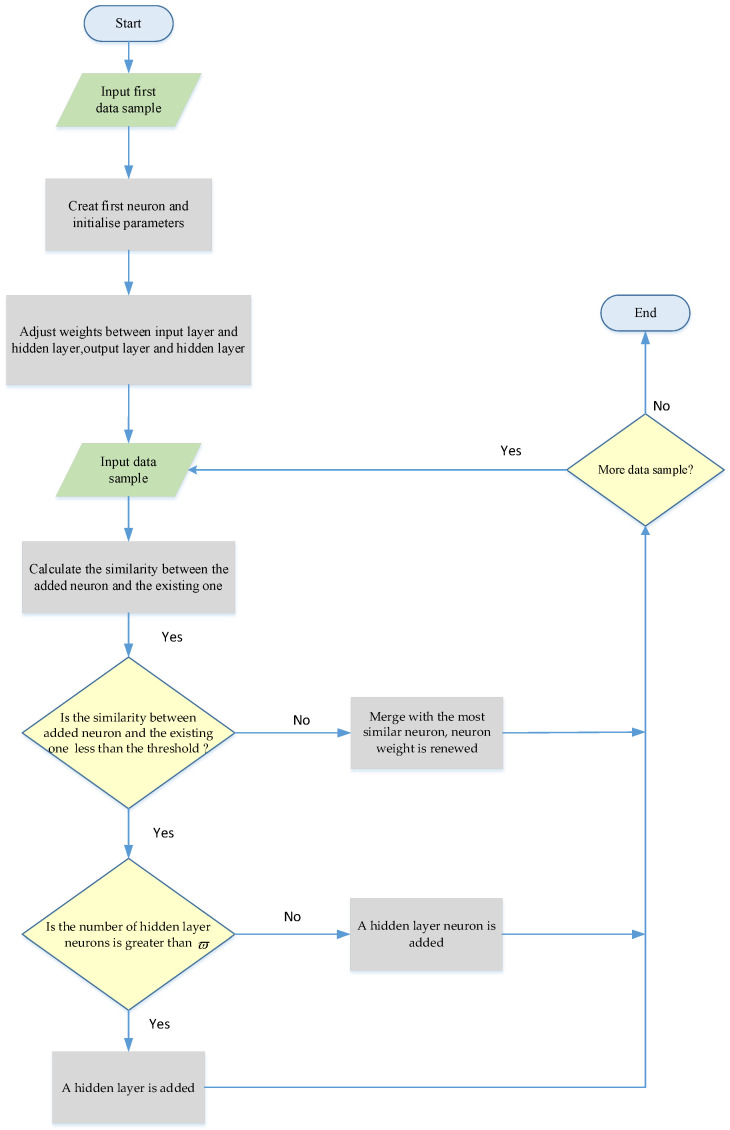
Flowchart of the dynamic cascade structure learning algorithm.

The dynamic structure learning algorithm is proposed to optimize the network size with hidden layers and the neuron number in each hidden layer. The flowchart of the proposed learning algorithm is described in Figure 5. One of the following three ways of network learning should be chosen for each input sample.

- A hidden-layer neuron is added. The derivative of the Euclidean distance is used to calculate the similarity between the added neuron and the existing one. If the similarity is less than the threshold ν, and the number of hidden-layer neurons is not greater than ϖ, this means that there is a big difference between the information in the network and the input sample, and new neurons are added to the hidden layer to record the new information;
- A hidden layer is added. If the similarity is less than ν and the number of hidden layer neurons is greater than ϖ, new neurons are added to a new hidden layer. This is because neurons in the same hidden layer have similar functions, and adding too many neurons will lead to repeated network training, which is not conducive to network stability. If the number of dynamic cascaded hidden layers exceeds the preset value, the network learning optimization is terminated to avoid catastrophic network expansion;
- The neuron weight is renewed. If the similarity is not less than ν, the new neuron will merge with the most similar neuron as follows:

(28) $$w_{q}=\frac{w_{q}\cdot N_{q}+w_{k}}{N_{q}+1}$$

with $N_{q}$ as the number of training samples.

### 4.3. System Stability Analysis

Due to the fact that the feedforward controller does not affect the stability of the system, the characteristic model based on the feedback controller is considered for the stability analysis of the twelve-rotor system, as shown in the following theorem.

**Theorem 1.**  *Taking the characteristic model of the twelve-rotor system in Equation (12) and the adaptive feedback controller in Equations (13)–(15) into consideration, if the condition in Equation (16) is met, the closed-loop system of the twelve-rotor UAV is stable*.

**Proof of Theorem 1.**  Substituting Equation (14) into Equation (12), we have

(29) $$x(k+1)=(1-qT)x(k)-\varepsilon T\left|x(k)\right|\arctan(x(k))+\Delta (k)$$

Then,

(30) $$\begin{array}{l} \left[x(k+1)-x(k)\right]\mathrm{sgn}(x(k))\\ =\left[-qTx(k)-\varepsilon T\left|x(k)\right|\arctan(x(k))+\Delta (k)\right]\mathrm{sgn}(x(k)\\ <-qT\left|x(k)\right|-\varepsilon T\left|x(k)\right|\arctan\left|x(k)\right|+\tau_{m}\\ <-\varepsilon T\left|x(k)\right|\arctan\left|x(k)\right|+\tau_{m}\\ <-\frac{\tau_{m}}{\left|x_{0}(k)\right|\arctan\left|x_{0}(k)\right|}\left|x(k)\right|\arctan\left|x(k)\right|+\tau_{m}\\ \leq 0 \end{array}$$

In addition,

(31) $$\begin{array}{l} x(k+1)x(k)\\ =\left[\left(1-qT)x(k)-\varepsilon T\left|x(k)\right|\arctan(x(k))+\Delta (k\right)\right]x(k)\\ >(1-qT-\frac{\pi }{2}\varepsilon T)x^{2}(k)+\Delta (k)x(k)\\ =(1-qT-\pi \varepsilon T)x^{2}(k)+\frac{\pi }{2}\varepsilon Tx^{2}(k)+\Delta (k)x(k) \end{array}$$

On account of the condition in Equation (16), it can be deduced that

(32) $$\begin{array}{l} x(k+1)x(k)\\ >(1-qT-\pi \varepsilon T)x^{2}(k)+\frac{\tau_{m}x^{2}(k)}{\left|x_{0}(k)\right|}+\Delta (k)x(k)\\ =(1-qT-\pi \varepsilon T)x^{2}(k)+\left|x(k)\right|\left\{\tau_{m}\frac{\left|x(k)\right|}{\left|x_{0}(k)\right|}+\Delta (k)\mathrm{sgn}(x(k))\right\}\\ \geq (1-qT-\pi \varepsilon T)x^{2}(k)+\left|x(k)\right|\left\{\tau_{m}\frac{\left|x(k)\right|}{\left|x_{0}(k)\right|}-\tau_{m}\right\}\\ =(1-qT-\pi \varepsilon T)x^{2}(k)\\ >0 \end{array}$$

Consequently, we find that $x(k+1)x(k)>0,\left[x(k+1)-x(k)\right]\mathrm{sgn}(x(k))<0,$ $\left[x(k+1)+x(k)\right]\mathrm{sgn}(x(k))>0$. The error can be guaranteed not to change sign during the convergence process, and the closed-loop system of the twelve-rotor UAV is stable. The proof of Theorem 1 is complete.

## 5. Numerical Simulation Results

Numerical simulations of the trajectory-tracking control for the twelve-rotor UAV are carried out to verify the effectiveness and robustness of the proposed composite strategy. The model parameters of the twelve-rotor UAV were taken from a prototype and are displayed in Table 1. The sampling period T was set to 0.01 s, and $G_{0}(k)$ was restricted to the scope of $0<N_{1}T^{2}\leq G_{0(i,j)}(k)\leq N_{2}T^{2}$ with $N_{1}=1$ and $N_{2}=100$. The forgetting factor λ was set to 0.996. The parameters of the DCSNN were configured as follows:$C_{m}=300\;\mathrm{pF},\tau_{L}=30\;nS,V_{T}=20\;mV,U_{res}=-70\;mV,\lambda_{L}=10\;ms$; the number of hidden layers was initially set to 1, and the hidden neuron threshold was ϖ=30.

The initial position of the twelve-rotor UAV in the scenario of the numerical simulation is described as ${ϒ}_{0}={\left[P_{x0},P_{y0},P_{z0},\phi_{0},\theta_{0},\psi_{0}\right]}^{T}={\left[8,0,0,0,0,0.1\right]}^{T}$. The desired trajectory is set as a butterfly-shaped geometric curve in three-dimensional space, which is given by

$$\left\{\begin{array}{c} P_{rx}=5+5\cos t/(1+\sin^{2}t)\\ P_{ry}=5\sin t\cos t/(1+\sin^{2}t)\\ P_{rz}=2 \end{array}\right.$$

A comparative trajectory-tracking control simulation between the proposed composite controller with the DCSNN and other existent methods such as the FSNN [37] and SNN [38] as feedforward controllers was implemented in the same simulation environment. FSNN used a fuzzy membership function, aiming to take advantage of the uncertainty of fuzzy logic and the bio-inspired properties of the spike neural network to improve the processing power for complex data. White noise with an amplitude of 0.15 Nm was applied as a lumped disturbance on the three position channels.

**Figure 6:**
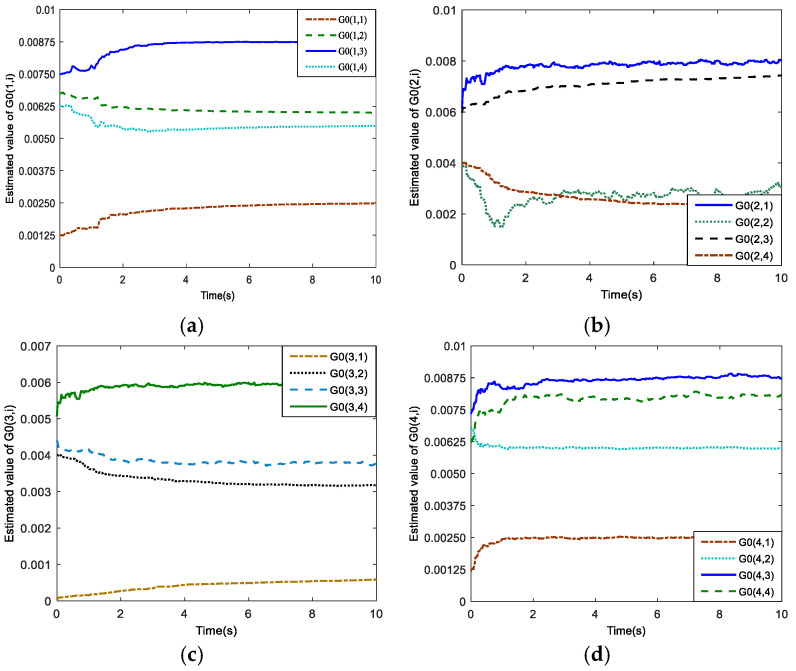
Estimation results of the characteristic parameter. (**a**) The first row; (**b**) the second row; (**c**) the third row; (**d**) the fourth row.

Figure 6 presents the estimation results for the characteristic parameter $G_{0}(k)$ based on the recursive least-square method with a forgetting factor. It can be observed that the estimated values eventually converge to a constant value at a fast convergence rate.

**Figure 7:**
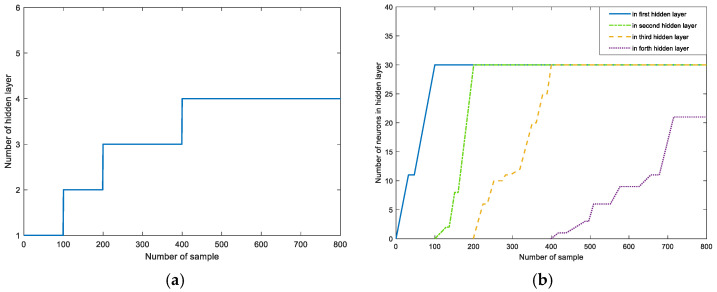
Evolution of the hidden-layer structure. (**a**) Number of hidden layers; (**b**) number of hidden-layer neurons.

The dynamic evolution processes of the hidden layers and hidden-layer neurons in the simulation are shown in Figure 7. The number of hidden layers converges to four, and the hidden neurons in each hidden layer are grown and pruned as the inputs tend to 30, 30, 30, and 22.

**Figure 8:**
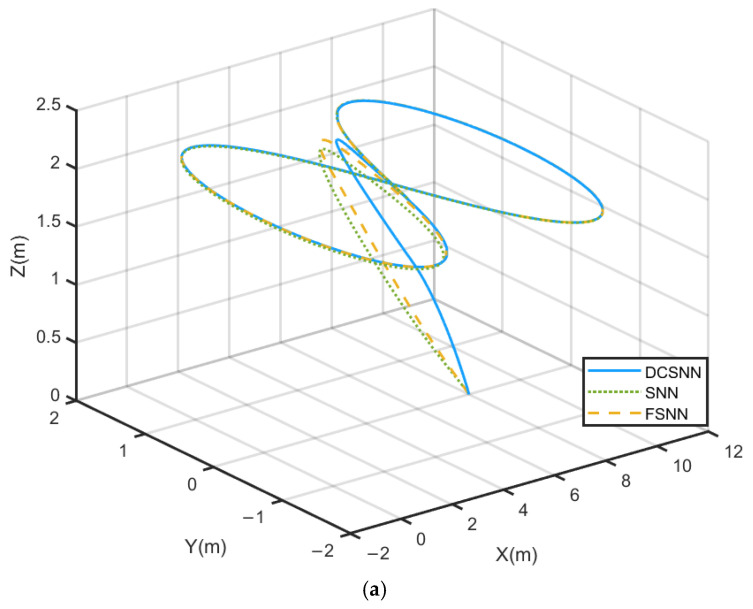

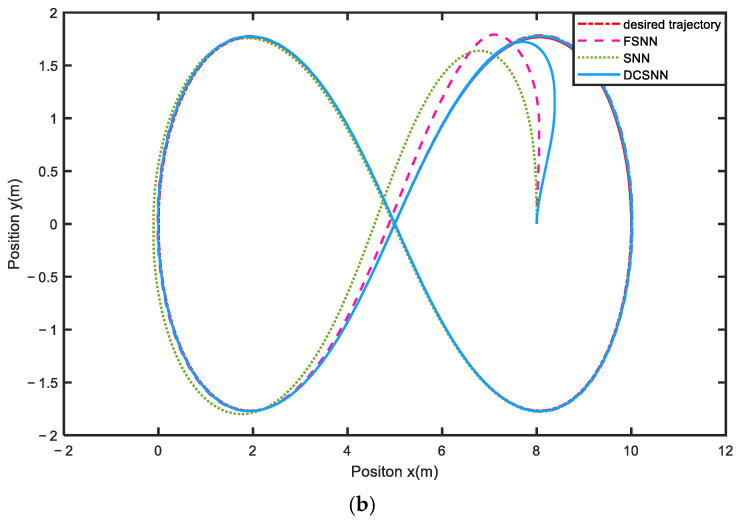
Comparison of the butterfly-shaped-curve-tracking results. (**a**) Three-dimensional space curve; (**b**) horizontal position curve.

**Figure 9:**
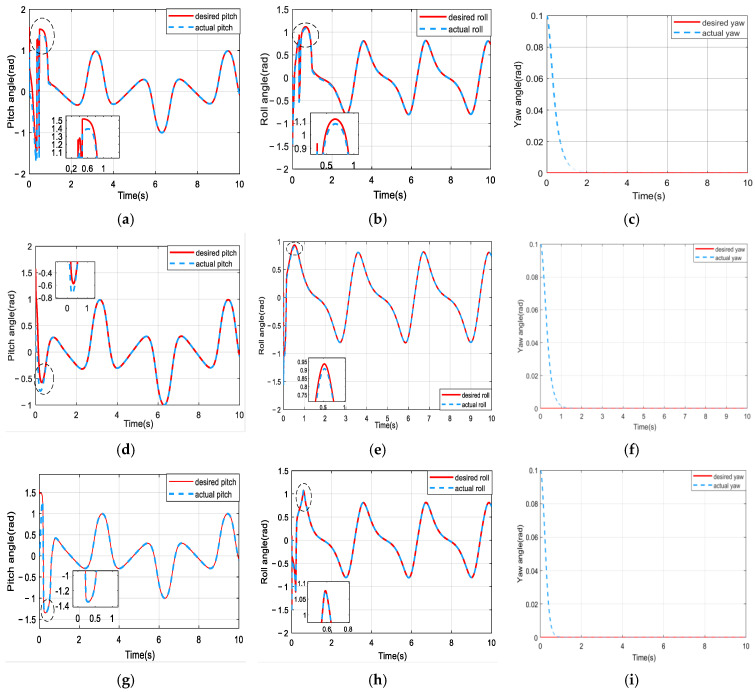
Comparison of the results for attitude performance. (**a**–**c**) SNN; (**d**–**f**) FSNN; (**g**–**i**) DCSNN.

**Figure 10:**
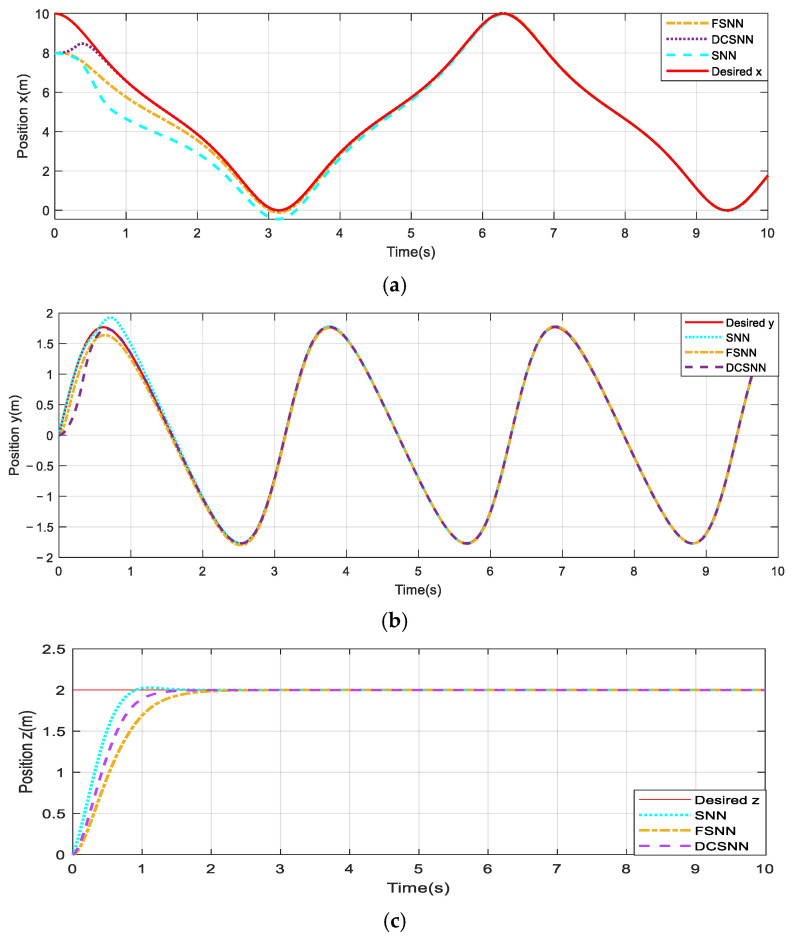
Comparison of the results for position performance. (**a**) Position x; (**b**) position y; (**c**) position z.

The comparative trajectory-tracking results are visualized in Figure 8. It is clear that the control performance of the proposed composite algorithm is significantly superior to that of the other two methods. The DCSNN displays a rapid altitude achievement, as shown in Figure 8a. In Figure 8b, the twelve-rotor UAV with the DCSNN tracks the desired trajectory at x=7.2 m,y=1.8 m. The DCSNN also responds faster and more accurately at the horizontal position. Furthermore, the comparative performance results for the attitude angles and position states are described in Figure 9 and Figure 10. It is worth pointing out that the proposed composite method with the DCSNN is more precisely convergent to the desired trajectories than the other two methods.

## 6. Twelve-Rotor Prototype Experiments

### 6.1. Experimental Setup

**Figure 11:**
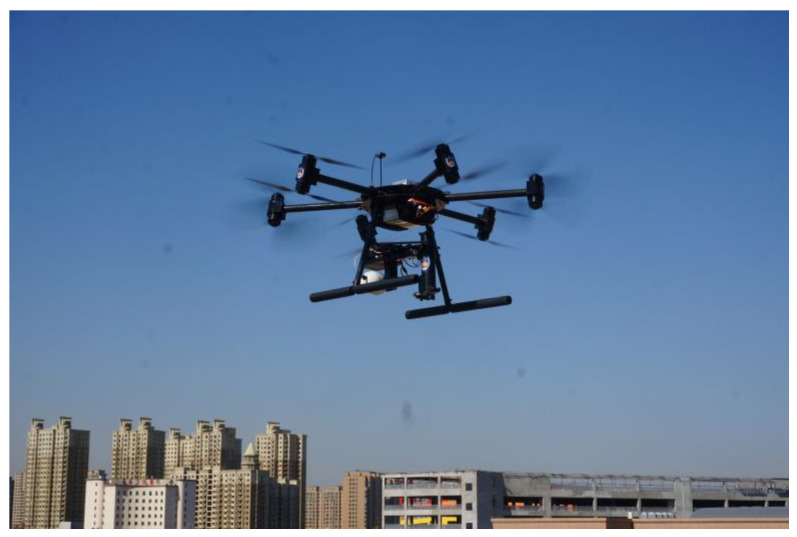
A photograph of the twelve-rotor prototype.

**Figure 12:**
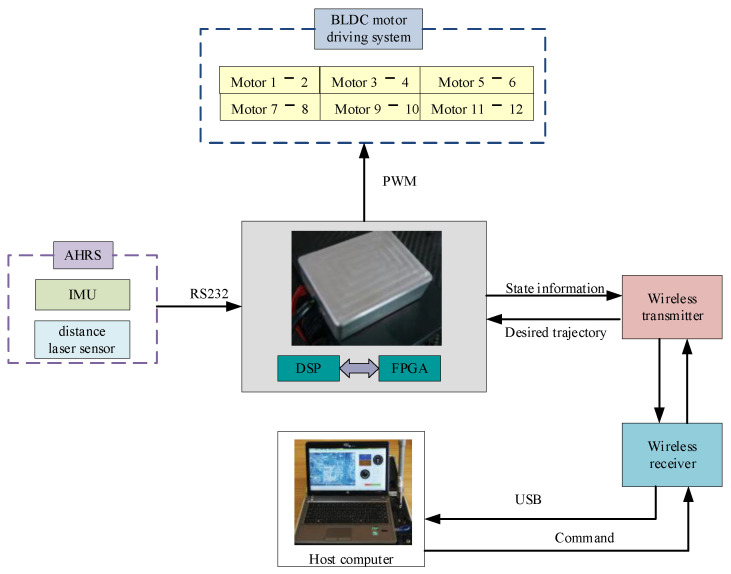
A schematic diagram of the control platform.

A photograph of the twelve-rotor prototype is exhibited in Figure 11. It is equipped with six pairs of blades driven by twelve brushless direct-current motors that are able to permit a flight duration of 40 min. Its maximum takeoff weight is about 15 kg. A schematic diagram of the control platform is depicted in Figure 12. It uses a combined strategy with Xilinx Zynq 7000 as a coprocessor and TMS320F28335 (DSP) as a master controller with a frequency of 150 MHz, thus fully meeting the requirements of real-time floating-point calculations of the composite control algorithm. The twelve-rotor flight states were measured using a distance laser sensor and inertial measurement unit (IMU), along with gyroscopes, accelerometers, and magnetometers. The accuracy of the distance laser sensor was ±1.5 mm. Furthermore, the flight control system received these flight data via an RS232 serial port. Meanwhile, the wireless transfer module was able to transmit flight states to the host computer, which was convenient for display and analysis. The host computer simultaneously generated the desired trajectories to command the twelve-rotor UAV.

### 6.2. Experimental Analysis

To verify its feasibility and superior tracking performance, comparative experiments on the adaptive sliding-mode algorithm based on the DCSNN and FSNN were performed in a windy outdoor environment on the twelve-rotor prototype. The parameters of the characteristic model and composite controller were set to the same values as those in simulations. The wind speed in the flight scenario measured with an anemometer was about 5 m/s, along with gusts. The twelve-rotor UAV was operated using a remote, and the desired trajectory was the rebound line.

**Figure 13:**
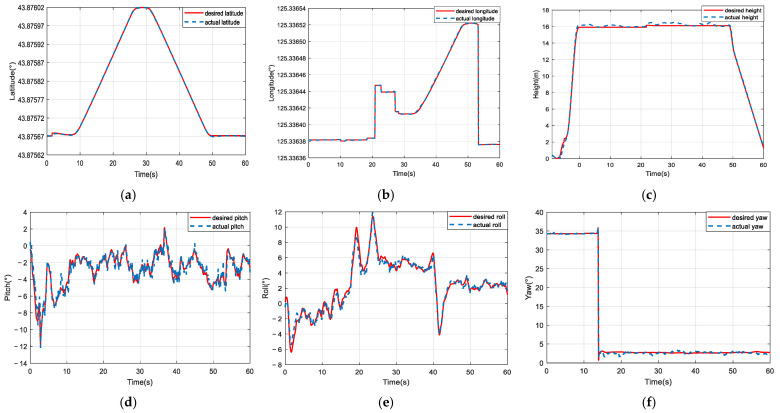
The results of the experiment on the twelve-rotor prototype based on the proposed DCSNN method. (**a**) Latitude-tracking curves; (**b**) longitude-tracking curves; (**c**) height-tracking curves; (**d**) pitch-angle-tracking curves; (**e**) roll-angle-tracking curves; (**f**) yaw-angle-tracking curves.

**Figure 14:**
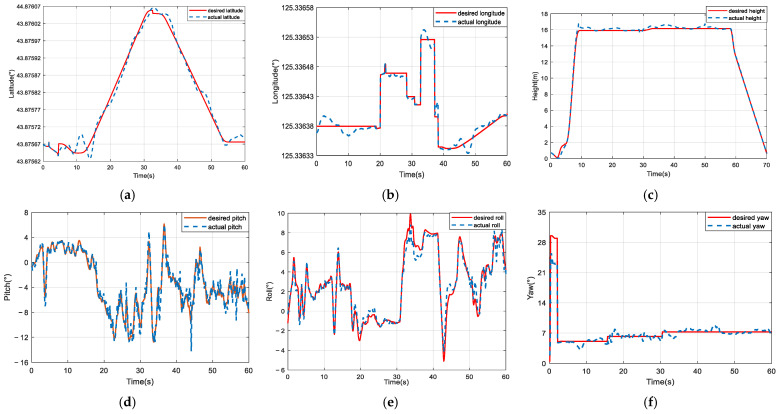
The results of the experiment on the twelve-rotor prototype based on the FSNN method. (**a**) Latitude-tracking curves; (**b**) longitude-tracking curves; (**c**) height-tracking curves; (**d**) pitch-angle-tracking curves; (**e**) roll-angle-tracking curves; (**f**) yaw-angle-tracking curves.

The prototype’s trajectory- and attitude-tracking results are portrayed in Figure 13 and Figure 14. It was observed that the twelve-rotor UAV based on the proposed composite controller had not only more favorable transient performance with smaller maximum tracking error fluctuation and a shorter settling time but also more satisfactory steady-state performance with smaller steady-state error and error fluctuation. The steady-state errors in the three trajectory directions based on the proposed composite method all converged within ±0.8 m in the presence of a level-three wind disturbance.

**Figure 15:**
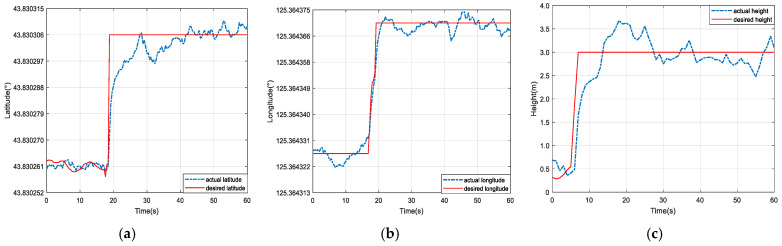
The results of the experiment on the twelve-rotor prototype based on the DCSNN method with a step-tracking signal. (**a**) Latitude-tracking curves; (**b**) longitude-tracking curves; (**c**) height-tracking curves.

**Figure 16:**
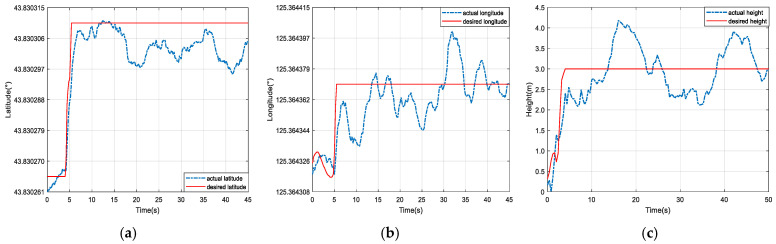
The results of the experiment on the twelve-rotor prototype based on the FSNN method with a step-tracking signal. (**a**) Latitude-tracking curves; (**b**) longitude-tracking curves; (**c**) height-tracking curves.

To further verify the control performance, the desired tracking trajectories in three directions were given as step signals. The control parameters were the same as those in the simulations. The instantaneous wind could reach 6 m/s in the flight experiment. The comparative prototype trajectory-tracking results between the DSCNN and FSNN are described in Figure 15 and Figure 16. Since the latitudinal motion was against the wind, the latitude direction based on the FSNN did not reach the desired trajectory, and there was a large steady-state error. Meanwhile, the longitudinal direction based on the FSNN had a significant error fluctuation due to the wind disturbance. Compared with this, the algorithm proposed in this study had better wind disturbance resistance, and the detailed control performance indices of the settling time, maximum steady-state error, and root mean square error are shown in Table 2. It is worth pointing out that the proposed composite strategy with the DCSNN had satisfactory trajectory-tracking control performance.

## 7. Conclusions

This study describes a novel intelligent composite controller for a twelve-rotor UAV that can realize high trajectory control performance in the presence of external disturbances, as demonstrated in a practical prototype flight. A characteristic model of the twelve-rotor is designed to create a decoupling difference model, which leads to a novel idea in the mathematical modeling of multi-rotor UAVs. An adaptive sliding-mode algorithm is used as a feedback controller and a new DCSNN is developed as a supervised feedforward controller to construct a composite control framework. The DCSNN solves the problem of SNNs requiring prior knowledge of network structure information and the sample proportion distribution. The dynamic cascade network structure makes the composite controller more suitable for variable environments through the dynamic adaptive addition of hidden layers and hidden-layer neurons. It can avoid catastrophic interference or forgetting in the face of new information. Even if the input data continuously change, this does not affect the normal operation of the network. A comparative numerical simulation and prototype experiment were carried out to validate the effectiveness and robustness of the proposed composite strategy. The twelve-rotor system has excellent trajectory-tracking control performance with a steady-state error converging to ±0.8 m and strong robustness against three levels of wind disturbances outdoors. The supervisory control method studied here has been applied to practical engineering tasks.

## Figures and Tables

**Table 1:** Parameters of the twelve-rotor prototype.

Parameters	Values
Mass m	4.5 kg
Distance between the rotor and the center l	0.5 m
Moment of inertia to the x $-\mathrm{axis}I_{x}$	$8.1\times 10^{-3}\;{\mathrm{Nm}/s}^{2}$
Moment of inertia to the y $-\mathrm{axis}I_{y}$	$8.1\times 10^{-3}\;{\mathrm{Nm}/s}^{2}$
Moment of inertia to the z $-\mathrm{axis}I_{z}$	$14.2\times 10^{-3}\;{\mathrm{Nm}/s}^{2}$
Angle between the rotor shaft and body plane γ	$30^{\circ }$
Thrust factor k	$54.2\times 10^{-6}\;{\mathrm{Ns}}^{2}$

**Table 2:** The results of the twelve-rotor prototype experiment with step signal control performance indices.

	Indices	Composite Method Based on the DCSNN			Composite Method Based on the FSNN		
Directions		Settling Time (s)	MSSM (m)	RMSE (m)	Settling Time (s)	MSSM (m)	RMSE (m)
Longitude		10.3	0.8	0.47	19.2	1.4	0.89
Latitude		8.7	0.6	0.21	10.8	1.5	0.96
Height		16.1	0.5	0.14	21.7	0.8	0.49

## Data Availability

The original contributions presented in this study are included in the article.
